# Differentially Expressed Genes and Enriched Signaling Pathways in the Adipose Tissue of Obese People

**DOI:** 10.3389/fgene.2021.620740

**Published:** 2021-05-19

**Authors:** Zhenhua Lu, Lingbing Meng, Zhen Sun, Xiaolei Shi, Weiwei Shao, Yangyang Zheng, Xinglei Yao, Jinghai Song

**Affiliations:** ^1^Department of General Surgery, Department of Hepato-Bilio-Pancreatic Surgery, Beijing Hospital, National Center of Gerontology, Institute of Geriatric Medicine, Chinese Academy of Medical Sciences, Beijing, China; ^2^Department of Cardiology, Beijing Hospital, National Center of Gerontology, Beijing, China; ^3^Key Laboratory of Environmental Nanotechnology and Health Effects, Research Center for Eco-Environmental Sciences, Chinese Academy of Sciences, Beijing, China; ^4^College of Resources and Environment, University of Chinese Academy of Sciences, Beijing, China

**Keywords:** adipose tissue, inflammation, bioinformatics, obesity, mechanism

## Abstract

As the prevalence of obesity increases, so does the occurrence of obesity-related complications, such as cardiovascular and cerebrovascular diseases, diabetes, and some cancers. Increased adipose tissue is the main cause of harm in obesity. To better understand obesity and its related complications, we analyzed the mRNA expression profiles of adipose tissues from 126 patients with obesity and 275 non-obese controls. Using an integrated bioinformatics method, we explored the functions of 113 differentially expressed genes (DEGs) between them. Gene ontology (GO) and kyoto encyclopedia of genes and genomes (KEGG) pathway enrichment analyses revealed that upregulated DEGs were enriched in immune cell chemotaxis, complement-related cascade activation, and various inflammatory signaling pathways, while downregulated DEGs enriched in nutrient metabolism. The CIBERSORT algorithm indicated that an increase in macrophages may be the main cause of adipose tissue inflammation, while decreased γδ T cells reduce sympathetic action, leading to dysregulation of adipocyte thermogenesis. A protein-protein interaction network was constructed using the STRING database, and the top 10 hub genes were identified using the cytoHubba plug-in in Cytoscape. All were confirmed to be obesity-related using a separate dataset. In addition, we identified chemicals related to these hub genes that may contribute to obesity. In conclusion, we have successfully identified several hub genes in the development of obesity, which provide insights into the possible mechanisms controlling obesity and its related complications, as well as potential biomarkers and therapeutic targets for further research.

## Introduction

Over the past 40 years, the incidence of obesity has increased in both developed and developing countries because of unbalanced diets, inadequate physical activity, chronic stress, and certain drugs and other environmental pollutants (Andersen et al., 2016; Yang et al., 2017; Sung et al., 2019; Tomiyama, 2019). According to the latest report of the World Health Organization, more than 1.9 million people were overweight in 2016, of which 6.5 million were obese (NCD Risk Factor Collaboration [NCD-RisC], 2017; WHO, 2018). Obesity can cause many chronic diseases, including cardiovascular and cerebrovascular diseases, diabetes, and some cancers of the colon, rectum, gastric cardia, liver, gallbladder, and pancreas (Arnold et al., 2016; Lauby-Secretan et al., 2016; Gadde et al., 2018). The most characteristic pathological changes associated with obesity are abnormal and excessive fat accumulation (WHO, 2018). Adipose tissue is widely distributed throughout the human body, and regulates metabolism by storing and releasing fatty acids and secreting adipokines (Giralt and Villarroya, 2013; Chait and den Hartigh, 2020). In obese people, this regulatory ability is disrupted, as is adipokine release (Munzberg and Myers, 2005; Leal and Mafra, 2013). Current treatment strategies for obesity includes inhibition of fat absorption, liposuction, and weight loss surgery (Yanovski and Yanovski, 2014; Apovian et al., 2015; Wolfe et al., 2016). However, these are symptomatic treatments rather than preventative measures controlling obesity development, and the number of obese individuals continues to increase annually. Therefore, it is important to identify biomarkers of obesity in adipose tissue and their related molecular mechanisms, to develop effective therapies to prevent obesity and its complications.

With the growing popularity of chip and sequencing technology, comprehensive bioinformatic analysis has become a promising method for exploring disease-related biomarkers and their molecular mechanisms (Sakharkar et al., 2019; Wan et al., 2020). For example, Zhang et al. found that the adipose tissues of insulin-resistant and non-insulin-resistant patients with obesity had differential expression of a number of genes, including matrix metallopeptidase 9, interleukin *(IL*)*6*, C-X-C motif chemokine ligand (*CXCL*) *8*, C-C motif chemokine ligand 4, and *CXCL10*, which are enriched for several functions, including cytokine-cytokine receptor interactions, tumor necrosis factor (TNF) signaling, and pathways in cancer (Zhang et al., 2019). Ida et al. found that ST8 alpha-N-acetyl-neuraminide alpha-2,8-sialyltransferase 2 expression in omental fat was significantly associated with weight loss after Roux-en-Y gastric bypass (Hatoum et al., 2013). In addition, circadian clock and metabolic gene expression rhythms are reduced in the subcutaneous adipose tissue of obese patients with type 2 diabetes compared to lean controls (Stenvers et al., 2019). However, no large-scale studies have been performed to examine the differential expression of genes in the adipose tissues of obese and non-obese populations. In this study, we used an integrative bioinformatics approach to analyze immune cell differences and gene expression differences in adipose tissue from obese and non-obese patients. Moreover, we combined the analysis of differentially expressed genes (DEGs) with functional enhancement and protein-protein interaction (PPI) analysis. Finally, we identified the hub genes that may contribute to obesity.

## Materials and Methods

### Research Design

**FIGURE 1:**
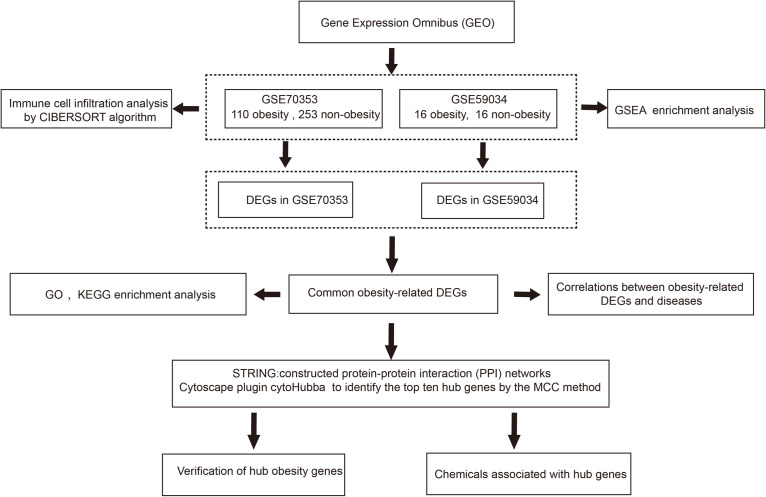
The flow chart of this study. DEGs, differentially expressed genes; GSEA, Gene set enrichment analysis; GO, Gene ontology; KEGG, Kyoto Encyclopedia of Genes and Genomes; MCC, maximal clique centrality.

The research was designed as shown in Figure 1. Firstly, we downloaded relevant mRNA expression profiles from the Gene Expression Omnibus (GEO). We then identified the pathways that may contribute to obesity through gene set enrichment analysis (GSEA) and analyzed the differences in immune cell infiltration in obese patients using the CIBERSORT algorithm. Secondly, we used an integrated bioinformatics approach to identify DEGs and explore the genes-related disease. Gene ontology (GO) and Kyoto Encyclopedia of Genes and Genomes (KEGG) pathway enrichment analyses were used to explore potential molecular mechanisms. A PPI network was constructed to identify hub genes. Finally, we verified the hub genes in a separate dataset and identified several chemicals that may contribute to obesity using the comparative toxicology genomics (CTD) database.

### Microarray Data

GEO^1^ is the largest public database of microarray- and sequencing-based expression data, and contains copious adipose tissue data (Edgar et al., 2002; Civelek et al., 2017). The search strategy used was “((obes^∗^) AND adipose tissue) AND “Homo sapiens”[porgn:_txid9606].” The inclusion criteria were as follows: subcutaneous adipose tissue samples, including those from obese [body mass index (BMI) > 30 kg/m^2^] and non-obese (BMI < 25 kg/m^2^) individuals. The GSE70353 and GSE59034 datasets were used. GSE70353 was submitted by the University of California, Los Angeles in 2015, and includes subcutaneous adipose biopsies from 770 people participating in the Metabolic Syndrome in Men study (Civelek et al., 2017). GSE59034 was submitted by the Karolinska Institute in 2014, and includes 48 subcutaneous adipose tissues from individuals with varying BMIs (Petrus et al., 2018), the details are presented in Table 1. After downloading the expression matrices and clinical data, samples were selected according to the inclusion criteria. Samples from 126 obese people (110 from GSE70353 and 16 from GSE59034) and 275 non-obese people (259 from GSE70353 and 16 from GSE59034) were used in the following analysis.

**TABLE 1:** Details for GEO obesity adipose tissue data.

**Dataset**	**Platform**	**Obese sample**	**Non-obese sample**	**Gender**	**Non-obese age (years)**	**Obese Age (years)**	**BMI for obesity (kg/m^2^)**	**BMI for non-obesity (kg/m^2^)**
GSE70353	GPL13667	110	259	Men	54.8 ± 4.9	54.8 ± 5.2	23.2 ± 1.3	32.6 ± 2.9
GSE59034	GPL11532	16	16	Women	47.7 ± 2.2	46.1 ± 2.1	25.1 ± 0.5	41.1 ± 0.9

*Age and BMI values are mean ± SEM (Standard Error of Mean); BMI: body mass index.*

### Immune Cell Analysis

CIBERSORT is an analytical tool that uses gene expression data to estimate the abundance of immune cell types in mixed cell populations (Newman et al., 2015). We used the original CIBERSORT gene signature file LM22, which defines 22 immune cell subtypes, to analyze datasets from obese adipose tissues and non-obese adipose tissues, with the number of permutations set at 100. The CIBERSORT *p*-value represents the statistical significance of the deconvolution results across all cell subsets and can be used to filter out deconvolution with less significant fitting accuracy. Samples with a *p* < 0.05 were selected from the calculated score table for further analysis. Then, the output was directly integrated to generate an entire matrix of immune cell fractions. The vioplot package (version 0.3.5) was used to visualize the results of immune infiltration between the adipose tissues of obese and non-obese subjects, and the Wilcoxon test was used to compare the differences in the means between obese and normal samples (Gehan, 1965).

### Gene Set Enrichment Analysis (GSEA)

GSEA was performed by comparing the obtained gene sets with known disease-related gene sets (Subramanian et al., 2005). GSE70353 and GSE59034 expression data and related phenotype files were imported into the GSEA software (version 4.0.2). The pre-validated curated C2 gene set database^2^ was employed as a reference to highlight the distinct biological features of obesity. Gene set permutations were performed 1,000 times for each analysis. A false discovery rate < 25% and a *p* < 5% were the criteria for significant gene set enrichment.

### DEG Identification

DEG identification was performed as follows. Firstly, to convert each probe expression matrix into a gene expression matrix, we downloaded the annotation platform file and then mapped the probe IDs to the gene symbols using R software (version 4.0.3). Probes without corresponding gene symbols were removed. For several probes that matched only one gene symbol, mean expression values were calculated and considered the final gene expression values. Then, DEGs in the obese and non-obese groups were analyzed using the linear models’ limma package (version 3.44.3), and the Benjamini–Hochberg method was used for multiple test correction. Genes with | log2 (fold change)| > 0.5, and a corrected *p* < 0.05 were considered DEGs. DEG heatmaps were generated using the Pheatmap package (version 1.0.12) (Kolde, 2012). Finally, the online tool Draw Venn Diagram^3^ was used to detect the DEGs shared by both datasets (Bardou et al., 2014). Common DEGs were used in the following analyses.

### Enrichment Analysis in DisGeNET

The DisGeNET database integrates multiple disease gene databases and is used to explore disease-related genes (Piñero et al., 2016). We used Metascape, an online tool that contains multiple enrichment methods (Zhou et al., 2019), to query DisGeNET to determine the proportion of obesity-related diseases.

### Pathway and Functional Enrichment Analyses

To identify the pathways in which DEGs cause obesity, we conducted GO and KEGG enrichment analyses. GO analysis involves detecting the enrichment of genes in particular cellular components, biological processes, and molecular functions (Gene Ontology Consortium[GOC], 2006). The KEGG database contains various biological pathways. KEGG enrichment analysis reveals which pathways DEGs are involved in, which can indicate the molecular mechanisms of a disease (Kanehisa and Goto, 2000). In this study, GO and KEGG enrichment analyses were both performed using the ClusterProfiler package (version 3.16.0) in R (with a *q* < 0.05 and a *p* < 0.05 considered significant) (Yu et al., 2012).

### PPI Network Construction and Hub Gene Identification

The STRING database (version 11)^4^ is a web resource containing all known and predicted PPI networks (Szklarczyk et al., 2016), and was used to explore the functional interactions between proteins. Based on the STRING online tool, PPI networks of the DEGs were constructed with a confidence score ≥ 0.4. After removing unconnected nodes from the network, DEG PPIs were visualized using Cytoscape (version 3.7.2). All data and information were exported and plotted using Cytoscape software. The DEGs are represented as nodes, and the interaction between DEGs are represented as edges between nodes. The plug-in cytoHubba was then used to identify hub genes in the network (Chin et al., 2014) and the top 10 hub genes were explored using the maximum clique centrality (MCC) method, a topological analysis method in CytoHubba for identifying feature nodes and hub genes from all DEGs.

#### Hub Gene Verification

To validate the differential expression of the hub genes in obesity, we used the GSE55200 dataset, which contains adipose tissue expression data from seven healthy lean people, eight metabolically healthy obese people, and eight metabolically unhealthy obese people (Badoud et al., 2014), by downloading its normalized gene expression matrix, converting the probes to gene names using R, and analyzing the differential expression of 16 obesity- and seven non-obesity-associated genes. Statistical analysis was performed using descriptive statistics and the two-tailed Student’s *t*-test in GraphPad Prism (version 8.0), with a *p* < 0.05 considered statistically significant (Mavrevski et al., 2018).

### Identification of Chemicals Associated With Hub Genes

The CTD database^5^ is a major public resource of literature-based, artificially planned linkages between chemistry, genetic products, phenotypes, diseases, and environmental exposure (Davis et al., 2019). We searched the database for the hub genes to identify chemicals associated with obesity and weight gain.

## Results

### Immune Cell Infiltration Analysis

**FIGURE 2:**
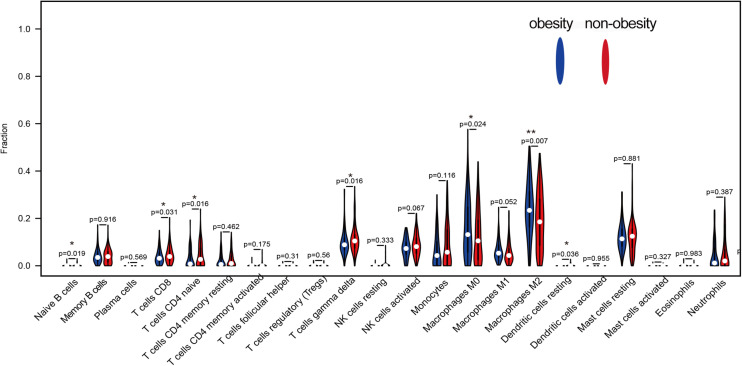
The difference of 22 kinds of immune cells in obesity and non-obesity, blue represents obese adipose tissue, red represents non-obesity adipose tissue.

CIBERSORT analysis of the expression matrix, which included samples from 93 obese people and 129 non-obese people who met the screening criteria (*p* < 0.05), was used to examine the immune cell contents. As shown in Figure 2, the immune cells in adipose tissue were mainly mast cells, T cells, macrophages, monocytes, and neutrophils. Compared with non-obese adipose tissue, M0 and M2 macrophages were significantly higher in obese adipose tissues (*p* < 0.05), while M1 macrophages were also higher, but the difference was not statistically significant (*p* = 0.052). However, naive CD4^+^, CD8^+^, and γδ T cells were significantly reduced in obese adipose tissues compared with non-obese tissues (*p* < 0.05).

### GSEA

**FIGURE 3:**
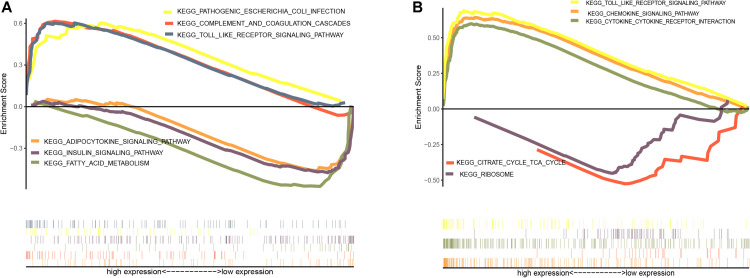
Enrichment analysis of gene set pathway. **(A)** Gene set enrichment analysis in GSE70353. **(B)** Gene set enrichment analysis in GSE59034.

GSEA was performed to identify possible mechanisms of obesity. The samples were divided into obese and non-obese groups. The analysis indicated that the most significantly enriched gene sets were positively correlated with the obesity group, and included pathogenic *Escherichia coli* infection, the complement and coagulation cascades, the toll-like receptor signaling pathway, the chemokine signaling pathway, and cytokine-receptor interactions (Figures 3A,B), while top gene sets negatively correlated with obesity were involved in fatty acid metabolism, the insulin signaling pathway, adipocytokine signaling pathway, the citric acid cycle, and the ribosome.

### Identification of Obesity-Related DEGs

**FIGURE 4:**
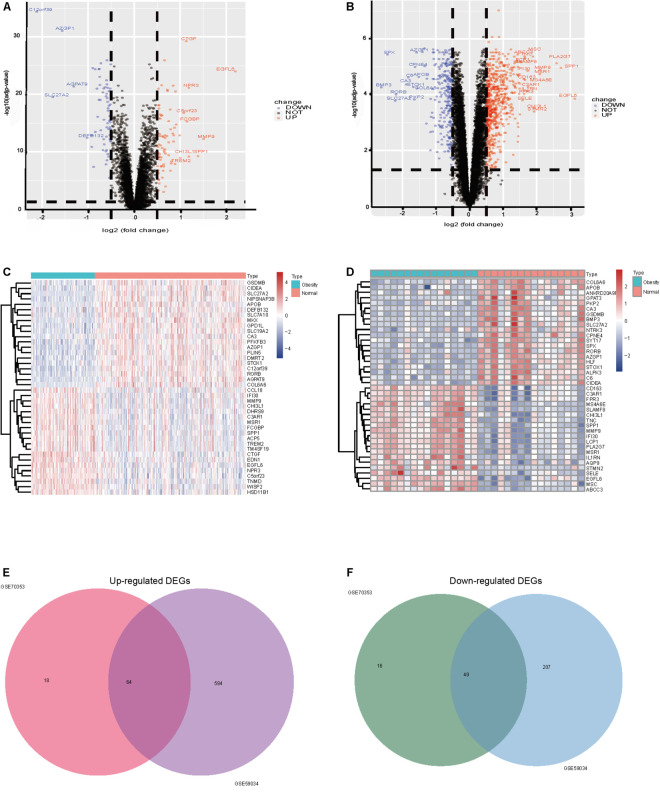
**(A)** GSE70353 volcano plots of differentially expressed genes, 82 upregulated and 65 downregulated, Upregulated genes are marked in red, downregulated genes are marked in blue; **(B)** GSE59034 volcano plots of differentially expressed genes 648 were upregulated and 256 downregulated; **(C)** GSE70353 top 20 up and down expressed genes heatmap, Upregulated genes are marked in red, downregulated genes are marked in blue; **(D)** GSE59034 top 20 up and down expressed genes heatmap; **(E)** up-regulation of co-expression Venn map with 64 up-regulation; **(F)** down-regulation of co-expressive Venn map with a total of 49 down-regulation co-expression.

After standardization and normalization of the microarray data, 147 and 904 DEGs between obese and non-obese adipose tissues were extracted from GSE70353 and GSE59034, respectively, as shown in the volcano plots (Figures 4A,B). GSE70353 included 82 upregulated genes and 65 downregulated genes, and GSE59034 included 648 upregulated genes and 256 downregulated genes. The heatmaps show the top 20 most significant downregulated and upregulated genes (Figures 4C,D). The two datasets shared 64 and 49 genes that were up- and downregulated in obesity (Figures 4E,F and Table 2), which are analyzed in later sections.

**TABLE 2:** Co-expression of differential genes.

**Type**	**Gene symbol**
Up	*EGFL6,MMP9,SPP1,NPR3,CHI3L1,FCGBP,CTGF,TREM2,ACP5,EDN1,WISP2,C3AR1,MSR1,DHRS9,TNMD,HSD11B1,CCL18,IFI30,CD163,ITGB2,UCHL1, TNFRSF11B,CCL19,VSIG4,LTBP2,CCND1,IL1RN,LYZ,IRF8,C1QC,PALLD,C1QB,CTSG,TYROBP,CD52,RNASE6,LCP1,CD68,NQO1,FCER1G,GLIPR2,CPVL, FCGR2B,SFRP4,VGLL3,SELP,PTGFR,FOLR2,CTSS,MSC,PLA2G7,SEMA3C,CD53,PLEK,SLIT2,CD248,CPA3,CCL13,GLIPR1,TFRC,ADAP2,CCL2, TUBB2A,TIMP1*
Down	*SLC27A2,AZGP1,APOB,SLC7A10,PFKFB3,CA3,DMRT2,COL6A6,CIDEA,MKX,STOX1,NIPSNAP3B,RORB,GPD1L,SLC19A2,GSDMB,MAP3K5,SLC19A3,C6,PHGDH,NDRG4,OR51E1,RASL10B,LDHD,GJC3,MYOC,BMP3,EIF4EBP1,TMEM100,CKB,NAALAD2,TWIST1,ORMDL3,GLUL,TTC36,ACSS3,ACVR1C,PKP2, DAPK2,FAT3,PMM1,LPIN1,GINS3,TTLL7,EPB41L4B,PXMP2,KLF15,GPR146,ALDH6A1*

### Correlations Between Obesity-Related DEGs and Diseases

Obesity-upregulated genes were related to many diseases, including liver cirrhosis, pulmonary fibrosis, asthma, hypertension, rheumatoid arthritis, diabetes, and allergies (Table 3).

**TABLE 3:** Analysis of differential genes and related diseases.

**GO**	**Description**	**Count**	**%**	**Log10 (P)**	**Log10 (q)**
C0023893	Liver cirhosis, experimental	20	31.00	–14.00	–10.00
C0034069	Pulmonary fibrosis	7	11.00	–8.70	–5.10
C0021368	Inflammation	7	11.00	–7.70	–4.30
C0020538	Hypertensive disease	9	14.00	–7.00	–3.90
C0006663	Calcinosis	5	7.80	–6.90	–3.80
C0004096	Asthma	6	9.40	−660	–3.60
C0003873	Rheumatoid arthritis	7	11.00	–6.40	–3.40
C0011853	Diabetes mellitus, experimental	6	9.40	–6.40	–3.40
C0022658	Kidney diseases	6	9.40	–6.20	–3.30
C0020517	Hypersensitivity	5	7.80	–6.20	–3.30
C0027626	Neoplasm invasiveness	6	9.40	–5.70	–2.80
C0018824	Heart valve disease	4	6.20	–5.60	–2.80
C0017661	IGA glomerulonephritis	4	6.20	–5.60	–2.70
C0023895	Liver diseases	5	7.80	–5.50	–2.70
C0032285	Pneumonia	5	7.80	–5.40	–2.70
C0003862	Arthralgia	5	7.80	–5.40	–2.60
C0151744	Myocardial ischemia	6	9.40	–5.20	–2.50
C0007621	Neoplastic cell transformation	5	7.80	–4.90	–2.20
C0016663	Pathological fracture	3	4.70	–4.70	–2.00
C0162820	Dermatitis, allergic contact	4	6.20	–4.60	–1.90

### GO Enrichment Analysis of DEGs

**FIGURE 5:**
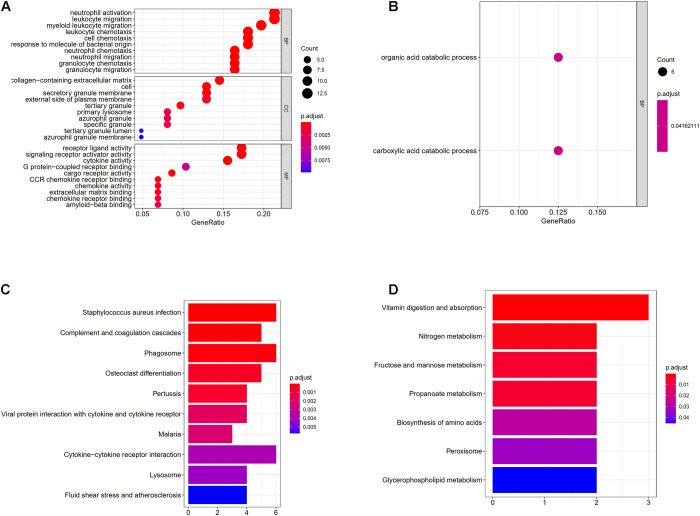
**(A)** Enrichment of upregulated differentially expressed genes in gene ontology (GO); **(B)** enrichment of downregulated differentially expressed genes in GO, different colored circles indicate different adjusted *P*-values. The size of the circle indicates the gene count. The *Y*-axis represents the GO term, the *X*-axis represents the gene proportion. **(C)** Enrichment of upregulated differentially expressed genes in KEGG, *X*-axis represents gene count, *Y*-axis represents different pathways, and different colors indicate different adjusted *P*-values; **(D)** enrichment of downregulated differentially expressed genes in KEGG.

The results of GO enrichment analysis varied with GO classification and DEG expression changes, as shown in Figures 5A,B. In terms of biological processes, upregulated DEGs were significantly enriched in myeloid leukocyte migration, neutrophil migration, granulocyte chemotaxis, granulocyte migration, leukocyte chemotaxis, cell chemotaxis, neutrophil activation, leukocyte migration, the response to molecules of bacterial origin, and chemokine-mediated signaling. For cellular components, DEGs were enriched in the secretory granule membrane, collagen-containing extracellular matrix, protein-lipid complexes, lipoprotein particles, plasma lipoprotein particles, and low-density lipoprotein (LDL) particles. Regarding molecular functions, upregulated DEGs were significantly enriched for cytokine activity, receptor-ligand activity, LDL particle binding, amide binding, and CCR chemokine receptor binding. However, in terms of biological processes, downregulated DEGs were only enriched in the carboxylic acid catabolic process and the organic acid catabolic process. More detailed GO enrichment results are shown in Supplementary Tables 1, 2. The number of upregulated genes involved in the different processes of BP, CC, and MF was much higher than that of down-regulated genes. These results suggest that obesity may undergo complex metabolic activities involving adipose tissue inflammation.

### KEGG Enrichment Analysis of DEGs

Upregulated genes were enriched in 23 pathways, and downregulated genes were enriched in seven pathways, as shown in Supplementary Tables 3, 4. The top 10 up- and downregulated pathways are shown in Figures 5C,D. Upregulated DEGs were enriched in pathways controlling *Staphylococcus aureus* infection, hypoxia-inducible factor 1 signaling, the complement and coagulation cascades, chemokine signaling, TNF signaling, and advanced glycation end products-receptor for advanced glycation end products signaling during diabetic complications, while downregulated genes were enriched in pathways controlling vitamin digestion and absorption, nitrogen metabolism, fructose and mannose metabolism, propanoate metabolism, amino acid biosynthesis, and glycerophospholipid metabolism.

### Construction of an Obesity-Related DEG PPI Network

**FIGURE 6:**
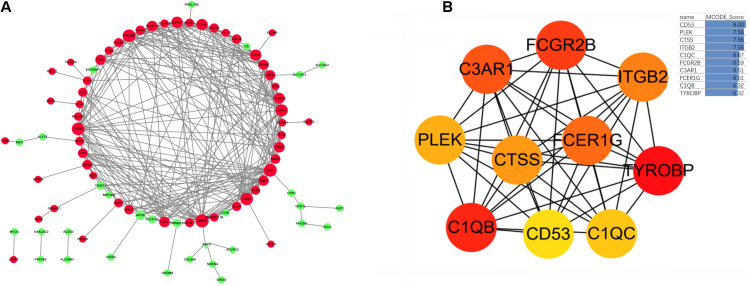
**(A)** The protein-protein interaction network diagram of DEGs in obese adipose tissue, red and green indicate that the node is upregulated and downregulated, respectively. The area of the node indicates the degree to which the node is connected to other nodes. **(B)** The top 10 hub genes screened by the MCC method in Cytohubba. The deeper yellow is the hub gene with a higher score, MCODE Score is calculated by MCC method, higher score means higher degree of connectivity.

Based on the STRING database, we developed a PPI network containing 85 nodes and 285 edges in the network. As shown in Figure 6A, nodes are drawn in different sizes and colors, representing the node degree and control (up or down), there were 30 downregulated genes, 55 upregulated genes and 285 edges. The MCC method in cytoHubba plug-in was used to identify the top ten hub genes (Figure 6B). These 10 hub genes were all upregulated genes, as follows: the CD53 molecule (*CD53*), pleckstrin (*PLEK*), cathepsin S (*CTSS*), integrin subunit beta 2 (*ITGB2*), TYRO protein tyrosine kinase binding protein (*TYROBP*), complement C1q B chain (*C1QB*), complement C1q C chain (*C1QC*), complement C3a receptor 1 (*C3AR1*), Fc fragment of IgG receptor IIb (*FCGR2B*), and Fc fragment of IgE receptor Ig (*FCER1G*).

### Verification of Hub Obesity Genes

**FIGURE 7:**
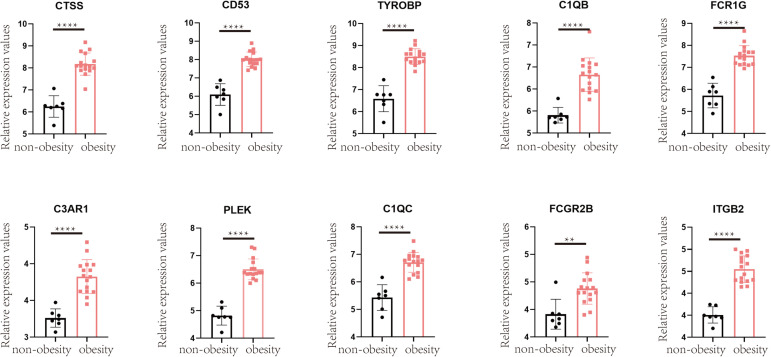
Verification in GSE55200 dataset, the comparison of hub gene in adipose tissue of obese and non-obese population. *Y*-axis represents the relative expression counts of genes. Obesity was marked in red, non-obesity was marked in black. ** for < 0.01, **** for < 0.0001.

To validate the changes in these hub genes in obesity, we performed relevant analyses in the GSE55200 dataset, which revealed that the expression of each hub gene was higher in the adipose tissue of obese people than in those of non-obese people (*p* < 0.05; Figure 7).

### Chemicals Associated With Hub Genes

We searched the CTD database for the 10 hub genes, and seven were related to obesity. For example, many chemicals have been identified that affect the expression of *CD53* and lead to obesity, including bisphenol A, cadmium chloride, carbon tetrachloride, choline, cisplatin, dexamethasone, dietary fats, diethylstilbestrol, ethinyl estradiol, lipopolysaccharides, methionine, parathion, phenobarbital, resveratrol, sodium selenite, streptozocin, tamoxifen, testosterone enanthate, tetrabromobisphenol A, tetrachlorodibenzodioxin, and tretinoin. The complete results are shown in Table 4.

**TABLE 4:** Chemicals associated with obesity and weight gain.

**Num.**	**Gene symbol**	**Gene name**	**Inference network**	**Inference score**
1	CD53	CD53 molecule	Benzo(a)pyrene, bissulfone, bisphenol A, Cadmium Chloride, Carbon Tetrachloride, Choline, Cisplatin, Dexamethasone, Dietary Fats, Diethylstilbestrol, Ethinyl Estradiol, Lipopolysaccharides, Methionine, Parathion, Phenobarbital, Resveratrol, Sodium Selenite, Streptozocin, Tamoxifen, testosterone enanthate, tetrabromobisphenol A, Tetrachlorodibenzodioxin, Tretinoin	76.60
2	FCER1G	Fc fragment of IgE receptor Ig	Azoxymethane, Benzo(a)pyrene, bis(4-hydroxyphenyl)sulfone, bisphenol A, Carbon Tetrachloride, Choline, Cisplatin, Cyclophosphamide, Dexamethasone, Dietary Fats, Diethylhexyl Phthalate, Diethylstilbestrol, Estradiol, Ethinyl Estradiol, Lipopolysaccharides, Methionine, methylmercuric chloride, Plant Preparations, Probucol, Quercetin, Resveratrol, sodium arsenite, Streptozocin, testosterone enanthate, tetrabromobisphenol A, Tetrachlorodibenzodioxin, Tobacco Smoke Pollution, Tretinoin, Troglitazone, Valproic Acid	101.45
3	TYROBP	TYRO protein tyrosine kinase binding protein	Acrylamide, benzo(a)pyrene, bissulfone, bisphenol a, carbon tetrachloride, choline, cisplatin, curcumin, cyclophosphamide, dexamethasone, dietary fats, diethylstilbestrol, estradiol, ethinyl estradiol, genistein, lipopolysaccharides, methionine, oxygen, resveratrol, sodium arsenite, streptozocin, tetrachlorodibenzodioxin, tobacco smoke pollution, tretinoin, troglitazone, valproic acid	84.36
4	CTSS	Cathepsin S	Bissulfone, bisphenol a, chlorpyrifos, cholesterol, dietary, curcumin, cyclosporine, dietary fats, diethylnitrosamine, fenretinide, folic acid, pirinixic acid, polychlorinated biphenyls, resveratrol, streptozocin, tetrachlorodibenzodioxin, valproic acid	37.38
5	C1QC	Complement C1q C chain	3,4,5,3′,4′-pentachlorobiphenyl, amitriptyline, azoxymethane, benzo(a)pyrene, bis(4-hydroxyphenyl)sulfone, bisphenol a, cadmium chloride, carbon tetrachloride, cisplatin, coenzyme q10, curcumin, cyclophosphamide, decabromobiphenyl ether, dexamethasone, dietary fats, diethylhexyl phthalate, diethylstilbestrol, estradiol, ethanol, ethinyl estradiol, genistein, lipopolysaccharides, lycopene, medroxyprogesterone acetate, nickel sulfate, oxygen, phenobarbital, resveratrol, sirolimus, streptozocin, tacrolimus, testosterone enanthate, tetrabromobisphenol a, tetrachlorodibenzodioxin, thioctic acid, tobacco smoke pollution, troglitazone, valproic acid	129.98
6	ITGB2	Integrin subunit beta 2	3,4,5,3′,4′-pentachlorobiphenyl, atorvastatin, azoxymethane, benzo(a)pyrene, bis(4-hydroxyphenyl)sulfone, bisphenol a, cadmium chloride, carbon tetrachloride, catechin, choline, cisplatin, decabromobiphenyl ether, dexamethasone, diazinon, dietary fats, environmental pollutants, estradiol, ethanol, ethinyl estradiol, lipopolysaccharides, lovastatin, methionine, probucol, quercetin, resveratrol, sodium arsenite, streptozocin, tetrabromobisphenol a, tetrachlorodibenzodioxin, tobacco smoke pollution, tretinoin, tributyltin, valproic acid	91.08
7	PLEK	Pleckstrin	Arsenic, azoxymethane, benzo(a)pyrene, bisphenol a, carbon tetrachloride, celecoxib, choline, cisplatin, dietary fats, ethanol, ethinyl estradiol, methionine, nickel sulfate, pioglitazone, plant extracts, quercetin, resveratrol, tetrabromobisphenol a, tetrachlorodibenzodioxin, Tobacco Smoke Pollution, Tretinoin	73.29

## Discussion

Globally, the incidence of obesity is continuously increasing. The harms and economic losses caused by obesity are significant. In 2014, economic losses directly or indirectly caused by obesity were as high as $2.0 trillion (Woetzel et al., 2014). The main feature of obesity is the accumulation of excessive adipose tissue, which can induce several diseases via oxygen deprivation, abnormal cytokine secretion, and interference with glycolipid metabolism (Chait and den Hartigh, 2020). Therefore, it is important to explore gene expression changes and associated molecular mechanisms in the adipose tissue of obese individuals. As the different estrogen levels and neural underpinnings affect body adipose tissue levels and appetite, obesity rates are higher in women than in men in most countries and regions, with an average prevalence of 10 and 18% in men and women, respectively (Kroll et al., 2020). Because of this, many studies have examined male and female obesity separately (Garawi et al., 2014). However, other studies have shown that variants of certain genes, including FTO alpha-ketoglutarate dependent dioxygenase, retinoic acid induced 1, MAGE family member L2, and melanocortin 4 receptor contribute to obesity development (Fall and Ingelsson, 2014; Poitou et al., 2020). To identify genes and pathways that cause obesity in everyone, in this study we analyzed two datasets, GSE70353, containing samples from men in Northern Finland with an average age of 54, and GSE59034, containing samples from women in Sweden with an average age of 45. We analyzed their DEGs separately, then performed overlap analysis to obtain a list of DEGs that were meaningful to all. A total of 64 upregulated and 49 downregulated genes were identified. Upregulated DEGs were associated with many diseases, including liver cirrhosis, pulmonary fibrosis, asthma, hypertension, rheumatoid arthritis, and diabetes. This evidence and epidemiological data provide mutual verification of the harmful effects of obesity (Chooi et al., 2019). These DEGs may represent key targets for the treatment of obesity and its complications.

Functional enrichment analyses revealed that the upregulated genes were mainly involved in the enhancement of inflammatory signals, including hypoxia-inducible factor 1, chemokine, and TNF signaling pathways. The increased oxygen consumption and the increasing size of adipocytes in turn leads to the occurrence of adipose tissue hypoxia (Lee et al., 2014). This may further induce increased production of adipokines such as chemokines, which increase myeloid leukocyte and neutrophil migration leading to chronic inflammation of adipose tissue (Krinninger et al., 2014). Downregulated genes were mainly involved in the metabolism of various nutrients, including fructose and mannose, propanoate, and glycerophospholipid metabolism, as well as amino acid biosynthesis (Shi et al., 2020). This indicates decreased metabolic function and nutrient dysregulation in the adipose tissue of obese people (Rogero and Calder, 2018). We also used GSEA to verify and supplement the DEG enrichment results. Pathways positively related to obesity included inflammatory infection, the complement cascade, and the toll-like receptor signaling pathway, which indicated that the complement system and toll-like receptors are the main factors of inflammation in adipose tissue during obesity (Rogero and Calder, 2018). Pathways negatively related to obesity included adipocytokine signaling, insulin signaling, and fatty acid metabolism, which reflect a dysregulation of adipokines and insulin signaling in obese patients. As enrichment analysis indicated inflammation-related signal upregulation in the adipose tissue of obese people, we used the CIBERSORT algorithm to detect whether there were changes in the immune cell composition between tissues from the two groups. It revealed increases in M0, M1, and M2 macrophages in the tissues of obese people, which may be the main reasons of adipose tissue inflammation. It also showed decreases in T cells, including CD4, CD8, and γδ types. Hu et al. showed that T cells (especially γδ T cells) can promote sympathetic innervation by driving the expression of transforming growth factor β1 in parenchymal cells via IL17 receptor C (Hu et al., 2020). Interestingly, recent studies have also reported fewer circulating γδ T cells in obesity (Li et al., 2020), which reduces the ability to fight viral infections, leading to a poorer prognosis for obese people than for lean people after infections such as severe acute respiratory syndrome coronavirus 2 (Jouan et al., 2020). Strategies to increase γδ T cell activation in these patients may be a promising direction for future research.

To identify the most critical hub genes among the 113 DEGs, we constructed a PPI network to identify the most interconnected genes. The top 10 hub genes were all upregulated. *CD53* is the most connected protein in the PPI network. It is mainly expressed on the membranes of immune cells, where it helps regulate many of their functions, including adhesion, migration, and cell fusion (Dunlock, 2020), and plays an important role in antigen presentation. Many studies have shown that *CD53* is increased in obese and inflammatory tissues, and regulating its expression may be an effective treatment for obesity with complications (Nair et al., 2005; Zhao et al., 2020). *PLEK* is the major protein kinase C substrate phosphorylated in diabetic macrophages, where a 30% reduction in PLEK can inhibit TNFα secretion by 80% (Ding et al., 2007). *CTSS* can promote adipocyte differentiation by degrading fibronectin. *In vitro* studies have also shown that the inflammatory factors TNFα and IL1β can induce cathepsin secretion in adipose tissue (Hooton et al., 2012). The expression of cathepsin mRNA in the adipose tissue of obese subjects was two times higher than that of healthy-weight subjects and decreased after weight loss, which shows that cathepsins can be effective markers of obesity (Naour et al., 2010).

*ITGB2* is positively correlated with obesity (Taleb et al., 2005; Pei et al., 2014; Imaizumi et al., 2018). It plays an important role in the immune response, and defects in *ITGB2* cause leukocyte adhesion deficiency (Ng et al., 2015). *TYROBP* is strongly related to insulin resistance and is mainly involved in the regulation of immune responses and integrin-mediated signaling (Das et al., 2015). *C3AR1* is a complement receptor that is expressed in both adipocytes and macrophages, and is increased by a high-fat diet (Mamane et al., 2009). *C3AR1* activation can enhance lipolysis induced by adrenaline, which may be a good way to treat obesity (Cero et al., 2017). *C1QB* and *C1QC* belong to a family of complement proteins that are positively correlated with immune cell chemotaxis and can regulate the expression of the immune cells. Recent findings have revealed that C1Q-related gene knockouts can prevent obesity-related complications of Alzheimer’s disease (Graham et al., 2020). *FCGR2B* and *FCER1G* are immunoglobulin fragments that regulate the immune response, and increase the likelihood of obese patients developing allergic diseases (Mathur et al., 2013). Shu et al. (2018) showed that FCGR2B promotes lipid accumulation and gluconeogenesis in hepatocytes.

In summary, *CD53, PLEK, CTSS, ITGB2, TYROBP, C1QB, C1QC, C3AR1, FCGR2B*, and *FCER1G* were all highly expressed in the adipose tissue of obese people. They all contribute to obesity and its complications, primarily through immune inflammation and glucolipid metabolism-related effects. In addition, the problem of environmental pollution is becoming increasingly serious, and many fat-soluble chemicals can accumulate in adipose tissue and interfere with gene expression, leading to obesity. The CTD database indicated that cadmium chloride, parathion, tetrachlorodiben, zodioxin, and bisphenol A may cause obesity by interfering with the expression of these hub genes. Therefore, it is important to reduce exposure to these chemicals. A limitation of this study is that it is strictly based on bioinformatic analysis, and the results will require further verification *in vitro* and *in vivo*.

## Conclusion

In conclusion, this is the first large-scale study to examine differential gene expression in the adipose tissues of obese and non-obese populations. Our study provides several potential biomarkers and related molecular mechanisms for obesity and its complications, and lays a foundation for the exploration of new targeted treatments.

## Data Availability Statement

The datasets presented in this study can be found in online repositories. The names of the repository/repositories and accession number(s) can be found in the article/ Supplementary Material.

## Author Contributions

ZL wrote the article. LM designed the study. ZS and XS were responsible for data collection and analysis. WS and YZ were responsible for data analysis and mapping. JS and XY revised and critically reviewed the article. All authors have agreed on the journal to which the article has been submitted, agreed to be accountable for all aspects of the work criteria, and made a significant contribution to the work reported.

## Conflict of Interest

The authors declare that the research was conducted in the absence of any commercial or financial relationships that could be construed as a potential conflict of interest.

## Footnotes

1 http://www.ncbi.nlm.nih.gov/geo

2 http://www.gsea-msigdb.org/gsea/msigdb/collections.jsp

3 http://jvenn.toulouse.inra.fr/app/example.html

4 http://www.string-db.org/

5 http://ctdbase.org/
