# Supplementary material for: Differentially Expressed Genes and Enriched Signaling Pathways in the Adipose Tissue of Obese People
Source: Front Genet. 2021 May 19;12:620740. doi: 10.3389/fgene.2021.620740 (PMC8175074; doi:10.3389/fgene.2021.620740)
Supplement: Supplementary file 1 [file Data_Sheet_1.docx]

Supplementary Material

# Supplementary table1. Up-regulated differential gene GO enrichment analysis

| ONTOLOGY | ID | Description | pvalue | p.adjust | qvalue | geneID |
| --- | --- | --- | --- | --- | --- | --- |
| BP | GO:0030593 | neutrophil chemotaxis | 1.32E-12 | 2.54E-09 | 1.79E-09 | CCL18/SLIT2/EDN1/CCL2/CCL19/FCER1G/IL1RN/C3AR1/ITGB2/CCL13 |
| BP | GO:0097529 | myeloid leukocyte migration | 3.21E-12 | 3.05E-09 | 2.16E-09 | CCL18/SLIT2/EDN1/CCL2/TREM2/CCL19/FCER1G/IL1RN/C3AR1/ITGB2/PLA2G7/CCL13 |
| BP | GO:1990266 | neutrophil migration | 4.75E-12 | 3.05E-09 | 2.16E-09 | CCL18/SLIT2/EDN1/CCL2/CCL19/FCER1G/IL1RN/C3AR1/ITGB2/CCL13 |
| BP | GO:0071621 | granulocyte chemotaxis | 7.22E-12 | 3.48E-09 | 2.46E-09 | CCL18/SLIT2/EDN1/CCL2/CCL19/FCER1G/IL1RN/C3AR1/ITGB2/CCL13 |
| BP | GO:0097530 | granulocyte migration | 2.84E-11 | 1.09E-08 | 7.74E-09 | CCL18/SLIT2/EDN1/CCL2/CCL19/FCER1G/IL1RN/C3AR1/ITGB2/CCL13 |
| BP | GO:0030595 | leukocyte chemotaxis | 1.43E-10 | 4.60E-08 | 3.25E-08 | CCL18/SLIT2/EDN1/CCL2/CCL19/FCER1G/IL1RN/C3AR1/ITGB2/PLA2G7/CCL13 |
| BP | GO:0060326 | cell chemotaxis | 3.61E-09 | 9.94E-07 | 7.03E-07 | CCL18/SLIT2/EDN1/CCL2/CCL19/FCER1G/IL1RN/C3AR1/ITGB2/PLA2G7/CCL13 |
| BP | GO:0042119 | neutrophil activation | 6.04E-09 | 1.33E-06 | 9.37E-07 | CTSG/LYZ/CHI3L1/FCGR2B/CTSS/TYROBP/FCER1G/C3AR1/CD53/CD68/ITGB2/GLIPR1/MMP9 |
| BP | GO:0050900 | leukocyte migration | 6.19E-09 | 1.33E-06 | 9.37E-07 | CCL18/SLIT2/SELP/EDN1/CCL2/TREM2/CCL19/FCER1G/IL1RN/C3AR1/ITGB2/PLA2G7/CCL13 |
| BP | GO:0002237 | response to molecule of bacterial origin | 1.26E-08 | 2.43E-06 | 1.72E-06 | CTSG/PTGFR/SELP/EDN1/CCL2/TREM2/FCGR2B/IL1RN/IRF8/CD68/ACP5 |
| BP | GO:0070098 | chemokine-mediated signaling pathway | 1.44E-08 | 2.52E-06 | 1.78E-06 | CCL18/SLIT2/EDN1/CCL2/TREM2/CCL19/CCL13 |
| BP | GO:0071674 | mononuclear cell migration | 1.69E-08 | 2.71E-06 | 1.91E-06 | CCL18/SLIT2/CCL2/CCL19/C3AR1/PLA2G7/CCL13 |
| BP | GO:0006959 | humoral immune response | 1.85E-08 | 2.75E-06 | 1.94E-06 | CTSG/C1QC/LYZ/CCL2/TREM2/C1QB/FCGR2B/C3AR1/VSIG4/RNASE6/CCL13 |
| BP | GO:1990868 | response to chemokine | 2.85E-08 | 3.66E-06 | 2.58E-06 | CCL18/SLIT2/EDN1/CCL2/TREM2/CCL19/CCL13 |
| BP | GO:1990869 | cellular response to chemokine | 2.85E-08 | 3.66E-06 | 2.58E-06 | CCL18/SLIT2/EDN1/CCL2/TREM2/CCL19/CCL13 |
| BP | GO:0043312 | neutrophil degranulation | 4.52E-08 | 5.45E-06 | 3.85E-06 | CTSG/LYZ/CHI3L1/CTSS/TYROBP/FCER1G/C3AR1/CD53/CD68/ITGB2/GLIPR1/MMP9 |
| BP | GO:0002283 | neutrophil activation involved in immune response | 4.84E-08 | 5.49E-06 | 3.88E-06 | CTSG/LYZ/CHI3L1/CTSS/TYROBP/FCER1G/C3AR1/CD53/CD68/ITGB2/GLIPR1/MMP9 |
| BP | GO:0002446 | neutrophil-mediated immunity | 6.17E-08 | 6.61E-06 | 4.67E-06 | CTSG/LYZ/CHI3L1/CTSS/TYROBP/FCER1G/C3AR1/CD53/CD68/ITGB2/GLIPR1/MMP9 |
| BP | GO:0002548 | monocyte chemotaxis | 6.72E-08 | 6.82E-06 | 4.82E-06 | CCL18/SLIT2/CCL2/CCL19/PLA2G7/CCL13 |
| CC | GO:0005623 | cell | 3.79E-10 | 4.74E-08 | 3.71E-08 | C1QC/LTBP2/CCL2/C1QB/TYROBP/CCL19/CD68/CCL13 |
| CC | GO:0030667 | secretory granule membrane | 4.13E-06 | 0.000196 | 0.000154 | SELP/TYROBP/FCER1G/C3AR1/CD53/CD68/ITGB2/GLIPR1 |
| CC | GO:0062023 | collagen-containing extracellular matrix | 4.71E-06 | 0.000196 | 0.000154 | CTSG/CPA3/C1QC/LTBP2/C1QB/EGFL6/CTSS/TIMP1/MMP9 |
| CC | GO:0070820 | tertiary granule | 1.26E-05 | 0.000395 | 0.000309 | LYZ/CTSS/FCER1G/CD53/ITGB2/MMP9 |
| CC | GO:0009897 | external side of plasma membrane | 3.06E-05 | 0.000766 | 0.0006 | FOLR2/SELP/FCGR2B/FCER1G/CD163/ITGB2/TFRC/CD248 |
| CC | GO:0005766 | primary lysosome | 0.000127 | 0.002266 | 0.001775 | CTSG/LYZ/C3AR1/CD68/GLIPR1 |
| CC | GO:0042582 | azurophil granule | 0.000127 | 0.002266 | 0.001775 | CTSG/LYZ/C3AR1/CD68/GLIPR1 |
| CC | GO:0042581 | specific granule | 0.000147 | 0.002301 | 0.001802 | LYZ/CHI3L1/C3AR1/CD53/ITGB2 |
| CC | GO:1904724 | tertiary granule lumen | 0.000691 | 0.009602 | 0.00752 | LYZ/CTSS/MMP9 |
| CC | GO:0035577 | azurophil granule membrane | 0.000808 | 0.009819 | 0.007689 | C3AR1/CD68/GLIPR1 |
| CC | GO:0034362 | low-density lipoprotein particle | 0.000864 | 0.009819 | 0.007689 | MSR1/PLA2G7 |
| CC | GO:0070821 | tertiary granule membrane | 0.001574 | 0.016401 | 0.012844 | FCER1G/CD53/ITGB2 |
| CC | GO:0005775 | vacuolar lumen | 0.002104 | 0.020232 | 0.015845 | CTSG/LYZ/CTSS/IFI30 |
| CC | GO:0005581 | collagen trimer | 0.002601 | 0.023221 | 0.018186 | C1QC/C1QB/MSR1 |
| CC | GO:0035579 | specific granule membrane | 0.002954 | 0.024621 | 0.019282 | C3AR1/CD53/ITGB2 |
| CC | GO:0002102 | podosome | 0.003999 | 0.03124 | 0.024466 | LCP1/PALLD |
| CC | GO:0034358 | plasma lipoprotein particle | 0.006036 | 0.04192 | 0.03283 | MSR1/PLA2G7 |
| CC | GO:1990777 | lipoprotein particle | 0.006036 | 0.04192 | 0.03283 | MSR1/PLA2G7 |
| CC | GO:0032994 | protein-lipid complex | 0.006689 | 0.044008 | 0.034465 | MSR1/PLA2G7 |
| MF | GO:0005125 | cytokine activity | 3.78E-08 | 6.66E-06 | 4.94E-06 | CCL18/EDN1/CCL2/CCL19/IL1RN/TNFRSF11B/TIMP1/SPP1/CCL13 |
| MF | GO:0048018 | receptor ligand activity | 3.31E-06 | 0.000213 | 0.000158 | CCL18/SEMA3C/EDN1/CCL2/CCL19/IL1RN/TNFRSF11B/TIMP1/SPP1/CCL13 |
| MF | GO:0030546 | signaling receptor activator activity | 3.63E-06 | 0.000213 | 0.000158 | CCL18/SEMA3C/EDN1/CCL2/CCL19/IL1RN/TNFRSF11B/TIMP1/SPP1/CCL13 |
| MF | GO:0038024 | cargo receptor activity | 4.91E-06 | 0.000216 | 0.00016 | FOLR2/CD163/ITGB2/MSR1/TFRC |
| MF | GO:0048020 | CCR chemokine receptor binding | 1.17E-05 | 0.00041 | 0.000304 | CCL18/CCL2/CCL19/CCL13 |
| MF | GO:0008009 | chemokine activity | 1.97E-05 | 0.000578 | 0.000429 | CCL18/CCL2/CCL19/CCL13 |
| MF | GO:0050840 | extracellular matrix binding | 3.61E-05 | 0.000906 | 0.000672 | SLIT2/CTSS/SPP1/CD248 |
| MF | GO:0042379 | chemokine receptor binding | 6.44E-05 | 0.001416 | 0.00105 | CCL18/CCL2/CCL19/CCL13 |
| MF | GO:0001540 | amyloid-beta binding | 0.000124 | 0.002419 | 0.001794 | TREM2/FCGR2B/ITGB2/MSR1 |
| MF | GO:0001664 | G protein-coupled receptor binding | 0.000302 | 0.005314 | 0.003941 | CCL18/EDN1/CCL2/CCL19/UCHL1/CCL13 |
| MF | GO:0019864 | IgG binding | 0.00057 | 0.009116 | 0.006761 | FCGR2B/FCER1G |
| MF | GO:0005539 | glycosaminoglycan binding | 0.000911 | 0.01244 | 0.009226 | CTSG/SLIT2/LTBP2/SELP/TREM2 |
| MF | GO:0005178 | integrin binding | 0.000919 | 0.01244 | 0.009226 | EGFL6/ITGB2/SPP1/LCP1 |
| MF | GO:0033218 | amide binding | 0.001066 | 0.012655 | 0.009386 | NPR3/FOLR2/TREM2/FCGR2B/ITGB2/MSR1 |
| MF | GO:0001846 | opsonin binding | 0.001079 | 0.012655 | 0.009386 | VSIG4/ITGB2 |
| MF | GO:0030169 | low-density lipoprotein particle binding | 0.00123 | 0.013531 | 0.010035 | TREM2/MSR1 |
| MF | GO:0001848 | complement binding | 0.00213 | 0.022053 | 0.016355 | VSIG4/ITGB2 |
| MF | GO:0008201 | heparin-binding | 0.00228 | 0.022295 | 0.016535 | CTSG/SLIT2/LTBP2/SELP |
| MF | GO:0005126 | cytokine receptor binding | 0.002425 | 0.022459 | 0.016657 | CCL18/CCL2/CCL19/IL1RN/CCL13 |
| MF | GO:0042277 | peptide-binding | 0.002771 | 0.023316 | 0.017291 | NPR3/TREM2/FCGR2B/ITGB2/MSR1 |

# Supplementary table2. Down-regulated differential gene GO enrichment analysis

| ONTOLOGY | ID | Description | pvalue | p.adjust | qvalue | geneID |
| --- | --- | --- | --- | --- | --- | --- |
| BP | GO:0016054 | organic acid catabolic process | 7.06E-05 | 0.041621 | 0.035265 | LPIN1/LDHD/SLC27A2/GLUL/TWIST1/ALDH6A1 |
| BP | GO:0046395 | carboxylic acid catabolic process | 7.06E-05 | 0.041621 | 0.035265 | LPIN1/LDHD/SLC27A2/GLUL/TWIST1/ALDH6A1 |

# Supplementary table3. UP-regulated differential gene KEGG enrichment analysis

| ID | Description | GeneRatio | pvalue | p.adjust | qvalue | genesymbol |
| --- | --- | --- | --- | --- | --- | --- |
| hsa05150 | Staphylococcus aureus infection | 6/42 | 9.13E-06 | 9.13E-06 | 0.000951 | C1QC,SELP,C1QB,FCGR2B,C3AR1,ITGB2 |
| hsa04610 | Complement and coagulation cascades | 5/42 | 7.30E-05 | 7.30E-05 | 0.003805 | C1QC,C1QB,C3AR1,VSIG4,ITGB2 |
| hsa04145 | Phagosome | 6/42 | 0.000123 | 0.000123 | 0.004273 | TUBB2A,FCGR2B,CTSS,ITGB2,MSR1,TFRC |
| hsa04380 | Osteoclast differentiation | 5/42 | 0.000499 | 0.000499 | 0.013012 | TREM2,FCGR2B,TYROBP,ACP5,TNFRSF11B |
| hsa05133 | Pertussis | 4/42 | 0.000626 | 0.000626 | 0.013054 | C1QC,C1QB,IRF8,ITGB2 |
| hsa04061 | Viral protein interaction with cytokine and cytokine receptor | 4/42 | 0.001749 | 0.001749 | 0.030374 | CCL18,CCL2,CCL19,CCL13 |
| hsa05144 | Malaria | 3/42 | 0.002185 | 0.002185 | 0.032521 | SELP,CCL2,ITGB2 |
| hsa04060 | Cytokine-cytokine receptor interaction | 6/42 | 0.003909 | 0.003909 | 0.049586 | CCL18,CCL2,CCL19,IL1RN,TNFRSF11B,CCL13 |
| hsa04142 | Lysosome | 4/42 | 0.004282 | 0.004282 | 0.049586 | CTSG,CTSS,CD68,ACP5 |
| hsa05418 | Fluid shear stress and atherosclerosis | 4/42 | 0.005736 | 0.005736 | 0.059453 | NQO1,EDN1,CCL2,MMP9 |
| hsa04614 | Renin-angiotensin system | 2/42 | 0.006276 | 0.006276 | 0.059453 | CTSG,CPA3 |
| hsa05323 | Rheumatoid arthritis | 3/42 | 0.012387 | 0.012387 | 0.105149 | CCL2,ACP5,ITGB2 |
| hsa05152 | Tuberculosis | 4/42 | 0.013973 | 0.013973 | 0.105149 | FCGR2B,CTSS,FCER1G,ITGB2 |
| hsa04933 | AGE-RAGE signaling pathway in diabetic complications | 3/42 | 0.015052 | 0.015052 | 0.105149 | CCND1,EDN1,CCL2 |
| hsa05142 | Chagas disease | 3/42 | 0.015869 | 0.015869 | 0.105149 | C1QC,CCL2,C1QB |
| hsa04062 | Chemokine signaling pathway | 4/42 | 0.016449 | 0.016449 | 0.105149 | CCL18,CCL2,CCL19,CCL13 |
| hsa05020 | Prion diseases | 2/42 | 0.018296 | 0.018296 | 0.105149 | C1QC,C1QB |
| hsa04066 | HIF-1 signaling pathway | 3/42 | 0.018922 | 0.018922 | 0.105149 | EDN1,TIMP1,TFRC |
| hsa05219 | Bladder cancer | 2/42 | 0.019171 | 0.019171 | 0.105149 | CCND1,MMP9 |
| hsa04668 | TNF signaling pathway | 3/42 | 0.020323 | 0.020323 | 0.105894 | EDN1,CCL2,MMP9 |
| hsa04650 | Natural killer cell-mediated cytotoxicity | 3/42 | 0.030506 | 0.030506 | 0.151382 | TYROBP,FCER1G,ITGB2 |
| hsa05322 | Systemic lupus erythematosus | 3/42 | 0.033559 | 0.033559 | 0.158965 | CTSG,C1QC,C1QB |
| hsa05416 | Viral myocarditis | 2/42 | 0.038891 | 0.038891 | 0.176212 | CCND1,ITGB2 |

# Supplementary table4. DOWN-regulated differential gene KEGG enrichment analysis

| ID | Description | GeneRatio | pvalue | p.adjust | qvalue | genesymbol |
| --- | --- | --- | --- | --- | --- | --- |
| hsa04977 | Vitamin digestion and absorption | 3/28 | 7.27E-05 | 7.27E-05 | 0.005051 | SLC19A3,SLC19A2,APOB |
| hsa00910 | Nitrogen metabolism | 2/28 | 0.001537 | 0.001537 | 0.053395 | CA3,GLUL |
| hsa00051 | Fructose and mannose metabolism | 2/28 | 0.005766 | 0.005766 | 0.106178 | PMM1,PFKFB3 |
| hsa00640 | Propanoate metabolism | 2/28 | 0.006113 | 0.006113 | 0.106178 | ACSS3,ALDH6A1 |
| hsa01230 | Biosynthesis of amino acids | 2/28 | 0.027022 | 0.027022 | 0.37547 | PHGDH,GLUL |
| hsa04146 | Peroxisome | 2/28 | 0.0334 | 0.0334 | 0.386741 | PXMP2,SLC27A2 |
| hsa00564 | Glycerophospholipid metabolism | 2/28 | 0.04519 | 0.04519 | 0.412646 | LPIN1,GPD1L |
